# Granulomonocytapheresis Using the Single-Needle Method for a Girl with Ulcerative Colitis

**DOI:** 10.1155/2021/5302885

**Published:** 2021-12-16

**Authors:** Yoshiaki Sasaki, Hiroki Kajino

**Affiliations:** Department of Pediatrics, Abashiri-Kosei General Hospital, Abashiri City, Hokkaido, Japan

## Abstract

Granulomonocytapheresis (GMA) is an effective treatment for inducing remission in patients with refractory ulcerative colitis (UC). Furthermore, GMA has very few side effects and can be performed without using drugs except anticoagulants. However, GMA is sometimes challenging to perform, especially in children, as it usually requires securing two blood vessels. We attempted GMA by the single-needle method in a girl with UC, which is performed by securing only one blood vessel. In the present case, GMA could be performed 10 times without any side effects. Our case shows that GMA with the single-needle method was feasible in children with UC.

## 1. Introduction

Granulomonocytapheresis (GMA) is a novel nonpharmacologic approach for treating irritable bowel disease using extracorporeal immunomodulation (cytapheresis). It is an effective treatment for inducing remission in patients with refractory ulcerative colitis (UC). Furthermore, GMA has very few side effects and can be performed without using drugs except anticoagulants. However, GMA is sometimes challenging to perform, especially in children, as it usually requires securing two blood vessels. To overcome this problem, we attempted GMA by the single-needle method, which is performed by securing only one blood vessel. Here, we report the case of a girl with UC who underwent GMA using the single-needle method followed by its assessment of feasibility for future use.

## 2. Case Presentation

**Figure 1:**
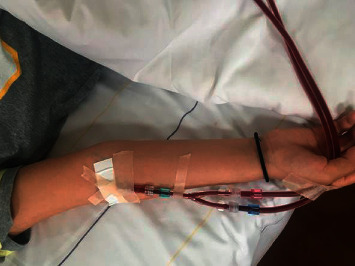
Granulomonocytapheresis with the single-needle method. A 17-gauge dialysis puncture needle (outer diameter: 1.4 mm, length: 25 mm) is inserted into the right elbow vein.

A 12-year-old girl developed abdominal pain and reported frequent bloody stools for over a month. She had been diagnosed with moderate left-sided UC at nine years of age. Remission was initially induced with prednisolone, and she remained in remission with azathioprine due to mesalazine intolerance. She experienced moon face and increased appetite as side effects while taking prednisolone. She was later diagnosed with a UC relapse based on colonoscopic findings of marked erythema and the absence of vascular pattern. Because of the side effects of previous prednisolone therapy, the patient and her guardian declined further steroid therapy. We decided to induce remission with GMA. However, securing two blood vessels for GMA was expected to be difficult because of the patient's small anthropometric measurement (height: 134.9 cm, weight: 31.7 kg). Therefore, we elected to perform GMA with the single-needle method. She underwent GMA once per week for 10 weeks. A 17-gauge dialysis puncture needle (outer diameter: 1.4 mm, length: 25 mm) was inserted into the right elbow (Figure 1). The dialysis console processed a blood flow rate of 40 mL/min (total blood volume: 1,800 mL). In this case, the treatment time was 90 minutes. No decrease in blood pressure was observed during this procedure. Heparin was used as an anticoagulant. All 10 GMA treatments were completed without puncture failure or poor blood removal. Additionally, no side effects were observed. However, the patient did not attain remission with GMA. After an unsuccessful attempt of oral tacrolimus therapy, remission could be achieved and has maintained with infliximab (5 mg/kg, every 8 weeks) for 10 months.

## 3. Discussion

Although treatments for UC include mesalazine, steroids, immunomodulators, and biologics, cytapheresis (including GMA) has also been reported as an effective therapeutic strategy [1, 2]. Disorders such as UC reflect overactive immune activation which is driven by cytokines derived from myeloid leukocytes (such as granulocytes and monocytes/macrophages). In GMA, cellulose acetate beads selectively adsorb and deplete myeloid cells and a small fraction of lymphocytes, thereby inducing anti-inflammatory effects [3]. Because GMA is a nonpharmacological treatment for UC, it is useful for children in whom the side effects of drugs, especially steroids, need to be avoided as much as possible. Although our patient developed mild dizziness because of the orthostatic dysregulation after the end of GMA, it appeared transiently and disappeared spontaneously. The incidence of adverse effects with GMA is significantly lower than that in conventional pharmacotherapy [4, 5].

**Figure 2:**
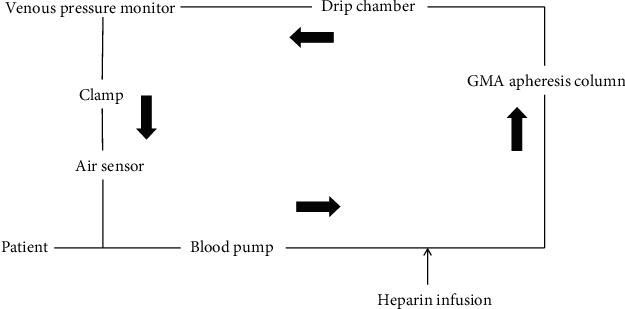
Circuit of granulomonocytapheresis with the single-needle method. Black arrow represents the blood flow.

When GMA is performed in children, securing the blood vessels is often a problem because of the small blood vessels. It is necessary to secure two blood vessels for blood removal and blood supply channels with conventional GMA. If it becomes difficult to secure blood vessels with this approach, conventional GMA must be abandoned. The single-needle method would become the alternative for performing GMA in such pediatric UC cases. In this method, blood removal and blood transfer are performed by securing only one blood vessel. In the blood removal process, the clamp closes and the blood pump rotates to remove blood. When the venous pressure in the circuit rises to 100 mm Hg, the blood pump stops, clamp is opened, and blood return process is started. When venous pressure in the circuit drops to 15 mm Hg, the blood removal process starts again (Figure 2). An advantage of the single-needle method is reduced psychological stress compared with the conventional method because the method results in a single blood vessel puncture. It also reduces the likelihood of an inability to puncture. Although a 17-gauge puncture needle did not result in poor blood removal, in this case, it is necessary to examine whether GMA can be performed with a smaller diameter puncture needle to reduce the burden on patients. In contrast, in the single-needle method, blood removal and blood transfer cannot be performed at the same time, so it takes longer to perform than conventional GMA, which takes approximately 60 minutes. In addition, the blood removal time should be monitored while paying attention to the decrease in blood pressure.

In our patient, GMA and TAC in 24 weeks were ineffective at inducing remission, and infliximab was required. Therefore, this case does not show the effectiveness of GMA.

The effectiveness of intensive GMA therapy that is administered several times a week has been previously reported [6, 7]. In case of a lack of response to infliximab, concomitant use with GMA should be considered to avoid surgery [4]. In addition, GMA is more effective in steroid-naive patients than in steroid-exposed patients [8]; therefore, an early introduction is desirable. In future, the single-needle method may become an effective procedure for performing GMA in children.

In conclusion, the single-needle method may potentially be a useful GMA method for treating UC in children in whom securing blood vessels is expected to be difficult.

## Data Availability

The data used to support the findings of this study are included within the article.
